# Incidents of violence and proportionality of restrictive practices: a D-FOREST study

**DOI:** 10.1192/j.eurpsy.2025.603

**Published:** 2025-08-26

**Authors:** J. Leahy, B. Walls, A. Lindner, D. Duffy, P. McLaughlin, H. G. Kennedy, M. Davoren

**Affiliations:** 1 National Forensic Mental Health Service, Dublin, Ireland; 2 University of Mainz, Mainz, Germany; 3 Department of Psychiatry, Trinity College Dublin, Dublin, Ireland

## Abstract

**Introduction:**

Aggression and violence are common in psychiatric in-patient wards. Preventive measures such as de-escalation, increased observations, extra medication, restraint and seclusion are utilised by nurses and authorised by doctors in a highly skilled way that should be proportionate to the risks posed. There is limited empirical data on the proportionality of the use of restrictive practices within Irish forensic psychiatric units.

**Objectives:**

The aim of this study was to rate the severity of incidents and proportionality of response to incidents in the high secure unit within the National Forensic Mental Health Service.

**Methods:**

This is a prospective cohort study set in the Central Mental Hospital. Patients were assessed each day using the Dynamic Assessment of Situational Aggression (DASA) scale and incidents were rated each day using the DUNDRUM Restriction-Intrusion of Liberty Ladders Scales (DRILL), which includes the assessment of adverse incidents, violence and self-harm, interventions including restrictive practices and consequences. In this study we used episodes of restriction as an outcome measure. Data were gathered as part of the Dundrum Forensic Redevelopment Evaluation Study (D-FOREST). Generalised Estimating Equations were used to analyse repeated measures in the same subjects.

**Results:**

There were 384 patient days in scope, 411 lines of data including 326 patient-days, 85 incidents and 63 incidents of seclusion. The DRILL scales had good internal consistency (DRILL behaviours scale Cronbach’s alpha=0.789; DRILL interventions scale Alpha=0.866). The DASA on the day before an incident predicted the score on the DRILL behaviours scale (severity of behaviours) Wald X^2^=39565.2, p<0.001, with DUNDRUM-1 triage security scale also contributing significantly to the model Wald X^2^=884.3, p<0.001. The best model to predict the DRILL interventions scale included DASA on the day before (Wald X^2^=14.6, p=0.012) DRILL-behaviours scale (Wald X^2^=728.7, p<0.001) and DUNDRUM-1 (Wald X^2^=10,819.4, p<0.001). This was also the best model to predict whether or not a patient was secluded (DASA day before Wald X^2^=46.4, p<0.001; DRILL-behaviours scale Wald X^2^=173.2, p<0.001; DUNDRUM-1 Wald X^2^=6153.5, p<0.001).

**Conclusions:**

Harmful behaviours and preventive and restrictive interventions can be described by rating items (‘ladders’) with good internal consistency, demonstrating that behaviour escalates in a meaningful sequence of increasingly serious harmful occurrences. The more serious the incident, the higher the level of restrictive practice used, demonstrating proportionality. This model of short term risk assessment and preventative interventions can be used to develop more effective and less restrictive interventions. Future research will explore moderating and mediating factors.

**Disclosure of Interest:**

None Declared

